# Age and Ovariectomy Abolish Beneficial Effects of Female Sex on Rat Ventricular Myocytes Exposed to Simulated Ischemia and Reperfusion

**DOI:** 10.1371/journal.pone.0038425

**Published:** 2012-06-06

**Authors:** Jenna L. Ross, Susan E. Howlett

**Affiliations:** 1 Department of Pharmacology, Dalhousie University, Halifax, Nova Scotia, Canada; 2 Division of Geriatric Medicine, Dalhousie University, Halifax, Nova Scotia, Canada; Ohio State University Medical Center, United States of America

## Abstract

Sex differences in responses to myocardial ischemia have been described, but whether cardiomyocyte function is influenced by sex in the setting of ischemia and reperfusion has not been elucidated. This study compared contractions and intracellular Ca^2+^ in isolated ventricular myocytes exposed to ischemia and reperfusion. Cells were isolated from anesthetized 3-month-old male and female Fischer 344 rats, paced at 4 Hz (37°C), exposed to simulated ischemia (20 mins) and reperfused. Cell shortening (edge detector) and intracellular Ca^2+^ (fura-2) were measured simultaneously. Cell viability was assessed with Trypan blue. Ischemia reduced peak contractions and increased Ca^2+^ levels equally in myocytes from both sexes. However, contraction amplitudes were reduced in reperfusion in male myocytes, while contractions recovered to exceed control levels in females (62.6±5.1 vs. 140.1±15.8%; p<0.05). Only 60% of male myocytes excluded trypan blue dye after ischemia and reperfusion, while all female cardiomyocytes excluded the dye (p<0.05). Parallel experiments were conducted in myocytes from ∼24-month-old female rats or 5–6-month-old rats that had an ovariectomy at 3–4 weeks of age. Beneficial effects of female sex on myocyte viability and contractile dysfunction in reperfusion were abolished in cells from 24-month-old females. Aged female myocytes also exhibited elevated intracellular Ca^2+^ and alternans in ischemia. Cells from ovariectomized rats displayed increased Ca^2+^ transients and spontaneous activity in ischemia compared to sham-operated controls. None of the myocytes from ovariectomized rats were viable after 15 minutes of ischemia, while 75% of sham cells remained viable at end of reperfusion (p<0.05). These findings demonstrate that cardiomyocytes from young adult females are more resistant to ischemia and reperfusion injury than cells from males. Age and OVX abolish these beneficial effects and induce Ca^2+^ dysregulation at the level of the cardiomyocyte. Thus, beneficial effects of estrogen in ischemia and reperfusion are mediated, in part, by effects on cardiomyocytes.

## Introduction

There are important sex-related differences in the pathophysiology of many cardiovascular diseases, including ischemic heart disease [1]–[6]. Clinical studies have established that pre-menopausal women are more resistant to ischemic heart disease than men of a similar age [7], but this female advantage disappears after the onset of menopause [8]. Furthermore, the risk of ischemic heart disease increases following bilateral ovariectomy, especially in women who have not taken exogenous hormone therapy [9]. It is well established that estrogen improves vascular function and reduces atherosclerosis, which may help reduce the risk of ischemic heart disease in younger women [10]. Beneficial effects of estrogen on cardiomyocytes themselves also may contribute, although this has not been extensively investigated [4], [11]. However, whether treatment with exogenous hormones is cardioprotective is controversial [12], [13], and a better understanding of mechanisms by which estrogen protects the heart in the setting of myocardial ischemia is needed.

Studies in animal models also have provided evidence for sex differences in responses to myocardial ischemia and reperfusion. Experiments in intact hearts from rats and mice have shown that young adult females exhibit better recovery of contractile function and fewer arrhythmias in reperfusion than age-matched males [14]–[20], although this has not been seen in all studies [21], [22]. Improved functional recovery in females is accompanied by smaller infarcts, less lactate dehydrogenase (LDH) release and less inflammatory cytokine production [23]–[25]; [17], [20]. Female hearts also exhibit less ischemia and reperfusion injury than males under conditions that promote Ca^2+^ loading, such as β-adrenergic stimulation or increased external Ca^2+^ [26], [27]. Even when sex differences are not observed in wild type hearts, female hearts show less ischemia and reperfusion injury in transgenic models with enhanced contractility, such as overexpression of Na^+^-Ca^2+^ exchanger [28], overexpression of β_2_-adrenergic receptors [29] or ablation of phospholamban [30]. Together, these observations indicate that ischemia and reperfusion injury is less severe in young adult females when compared to males. However, these studies used intact hearts, where estrogen receptors in both cardiomyocytes and the vasculature may modify responses to ischemia and reperfusion [11]. Whether responses of individual cardiomyocytes to myocardial ischemia is influenced by sex and whether this can help explain the resistance of female hearts to ischemia and reperfusion injury has not been investigated.

We have developed a model of ischemia and reperfusion injury in ventricular myocytes isolated from the hearts of male animals [31]–[33]. This model uses a simulated “ischemic” Tyrode’s solution that mimics features of ischemia such as hypoxia, acidosis, lactate accumulation, hyperkalemia, hypercapnia, and substrate deprivation. When myocytes from male animals are exposed to simulated ischemia they exhibit increased intracellular Ca^2+^ levels, along with post-ischemic contractile dysfunction (stunning) and reduced viability in reperfusion [32]–[33]. The objectives of this study were to use this model to determine: 1) whether ventricular myocytes isolated from young adult female hearts were resistant to ischemia and reperfusion injury when compared to cells from age-matched males; 2) whether ischemia and reperfusion injury was exacerbated by the aging process in myocytes from aged females; and 3) whether cellular ischemia and reperfusion injury was enhanced by long term reduction in ovarian estrogen induced by ovariectomy (OVX). Contractile function, intracellular Ca^2+^ and cell viability were compared throughout ischemia and reperfusion in ventricular myocytes from 3-month-old rats of both sexes. Some studies also used myocytes from aged (∼24-month-old) female rats and myocytes from 5–6-month-old female rats who had undergone a bilateral OVX at 3–4 weeks of age. Our results demonstrated that individual ventricular myocytes from young adult females were significantly more resistant to ischemia and reperfusion injury than cells from age-matched males. This female advantage was abolished by either advanced age or by removal of ovarian estrogen through OVX. These data indicate that beneficial actions of estrogen in myocardial ischemia are mediated, in part, by actions on the myocytes themselves.

## Materials and Methods

For full details of Methods, please refer to Appendix S1.

### Ethics Statement

Protocols were approved by the Dalhousie University Committee on Laboratory Animals (No. 10-029) and followed Canadian Council on Animal Care Guide to the Care and Use of Experimental Animals (CCAC, Ottawa, ON: Vol, 1, 2nd edition, 1993: Vol. 2, 1984). Sodium pentobarbital anesthesia was used and all efforts were made to reduce suffering.

### Myocyte Isolation

Myocytes were isolated from 3 month old male and female Fischer 344 rats, ∼24 month old females and from 5–6 month old females after OVX or sham operation at 3–4 weeks. Ventricular myocytes were isolated as described [33]. Briefly, rats were anesthetized with sodium pentobarbital (220 mg/kg, IP). The heart was perfused through the aorta with oxygenated low Ca^2+^ buffer (200 µM) followed by nominally Ca^2+^ free buffer plus collagenase and dispase II (37°C). After digestion, the ventricles were minced, stored in a high K^+^ substrate-enriched buffer and filtered before use. OVX was confirmed by uterine atrophy.

### Ischemia and Reperfusion

Myocytes were loaded with fura-2 AM as described [33] and superfused at 37°C with normal Tyrode’s solution (in mM: 126 NaCl, 20 NaHCO_3_, 0.9 NaH_2_PO_4_, 4 KCl, 0.5 MgSO_4_, 1.8 CaCl_2_, 5.5 glucose; pH 7.4, 95% O_2_, 5% CO_2_). Cells were paced at 4 Hz with trains of 20 (3 ms) pulses followed by a 2.5 s delay. The pacing frequency of 4 Hz was chosen to be near the physiological frequency in the rat. The 2.5 s pauses were incorporated in the protocol to observe spontaneous activity if it occurred. Control recordings were made for 15 minutes in normal Tyrode’s solution, then cells were exposed ischemic Tyrode’s solution for 20 minutes (in mM: 123 NaCl, 6 NaHCO_3_, 0.9 NaH_2_PO_4_, 8 KCl, 0.5 MgSO_4_, 20 Na-Lactate, and 1.8 CaCl_2_; pH 6.8, 90% N_2_, 10% CO_2_) [31]. During ischemia, 90% N_2_, 10% CO_2_ was directed over the experimental chamber to reduce the *p*O_2_ as described previously [34]. Cells were reperfused with normal Tyrode’s solution for up to 30 min. Cell that exhibited hypercontracture, sarcolemmal disruption and trypan blue staining were considered not viable [35]. Recordings were made at 5 min intervals throughout the protocol, with an additional recording at 2 min of reperfusion. Time controls were exposed to normal Tyrode’s solution for 65 min without ischemia.

Contractions and Ca^2+^ transients were recorded simultaneously as described [33]. Ca^2+^ levels were recorded with a DeltaRam fluorescence recording system (Photon Technology International (PTI), Birmingham, NJ) and data were acquired with Felix32 software (PTI). Fura-2 was excited at 340 and 380 nm and emission was measured at 510 nm (5 msec sampling interval). Unloaded cell shortening was measured (120 Hz) with a video edge detector (Model 105; Crescent Electronics, Sandy, UT) and CCD camera (model TM-640, Pulnix America). The ratio of fluorescence at 340 and 380 nm was converted to Ca^2+^ concentration as described previously [32], [33]. Results are accurate over the range of pH values used in this study as reported previously [36] and confirmed in our earlier studies [32], [33].

### Analyses

Data were analyzed with Clampfit 8.2 software (Molecular Devices). The last three responses in the 20 pulse train were averaged to quantify contractions and Ca^2+^ transients once these responses had reached steady state. The incidence of spontaneous activity (beats that occurred during the 2.5 s pause) was recorded in each experiment. Alternans (alternating pattern of large and small beats during stimulation) were quantified with an alternans ratio (alternans ratio = 1 – S/L, where S  =  amplitude of small beat and L =  amplitude of large beat) [33]. The value of "n" is the number of myocytes used. Statistical analyses were performed with either Sigmaplot 8.1 or Sigmastat 3.1 (Systat Software Inc.). Data other than cell viability and incidence are presented as the mean ± SEM. Cell viability was evaluated with a log rank test. Spontaneous activity was analyzed with a Fisher Exact test. Other analyses used a t-test or a two-way repeated-measures analysis of variance (post-hoc test = Student-Newman-Keuls or Tukey tests). Differences were significant for p<0.05.

## Results

### Physical Characteristics of Young Adult Male and Female Rats

Selected physical characteristics of young adult male and female rats were compared as shown in Table 1. Males and females were the same age, but the males were 67% heavier than the females (Table 1). Table 1 also shows baseline peak contractions and Ca^2+^ transients recorded from male and female myocytes after 15 minutes of pacing at 4 Hz, just prior to exposure to simulated ischemia and reperfusion. Contractions were normalized to cell length, as we found that cells from females were smaller than cells from males (Table 1). Peak contractions and Ca^2+^ transients were similar in cells from males and females under these experimental conditions (Table 1). Systolic and diastolic Ca^2+^ levels also were similar in all groups (not shown).

**Table 1:** Baseline Characteristics: Experiments with Young Adult and Aged Rats.

Parameter	Young Male(n)	Young Female (n)	Aged Female (n)
Age (days)	90.3±2.9† (15)	93.2±5.0† (13)	744.3±3.7 (11)
Body weight (g)	300.4±6.2 (15)	179.2±3.1* † (13)	330.4±12.8 (11)
Myocyte length (µm)	112.5±3.9 (16)	92.6±3.6* † (12)	121.3±4.7 (12)
Contraction (%)	2.7±0.6 (13)	2.6±0.5 (10)	2.4±0.4 (8)
Ca^2+^ transient (nM)	102.3±7.9 (12)	97.0±7.0 (10)	81.3±6.8 (10)

Values shown are the means ± SEM. The value of "n" shown beside each number in brackets represents the number of animals or myocytes. Values represent peak contractions (expressed as % cell length) and Ca^2+^ transients recorded prior to ischemia in myocytes paced at 4 Hz.

* Denotes significantly different from young male animal p<0.05.

† Denotes significantly different from aged female animal, p<0.05.

### Responses of Isolated Ventricular Myocytes to Simulated Ischemia and Reperfusion Differ between the Sexes

**Figure 1:**
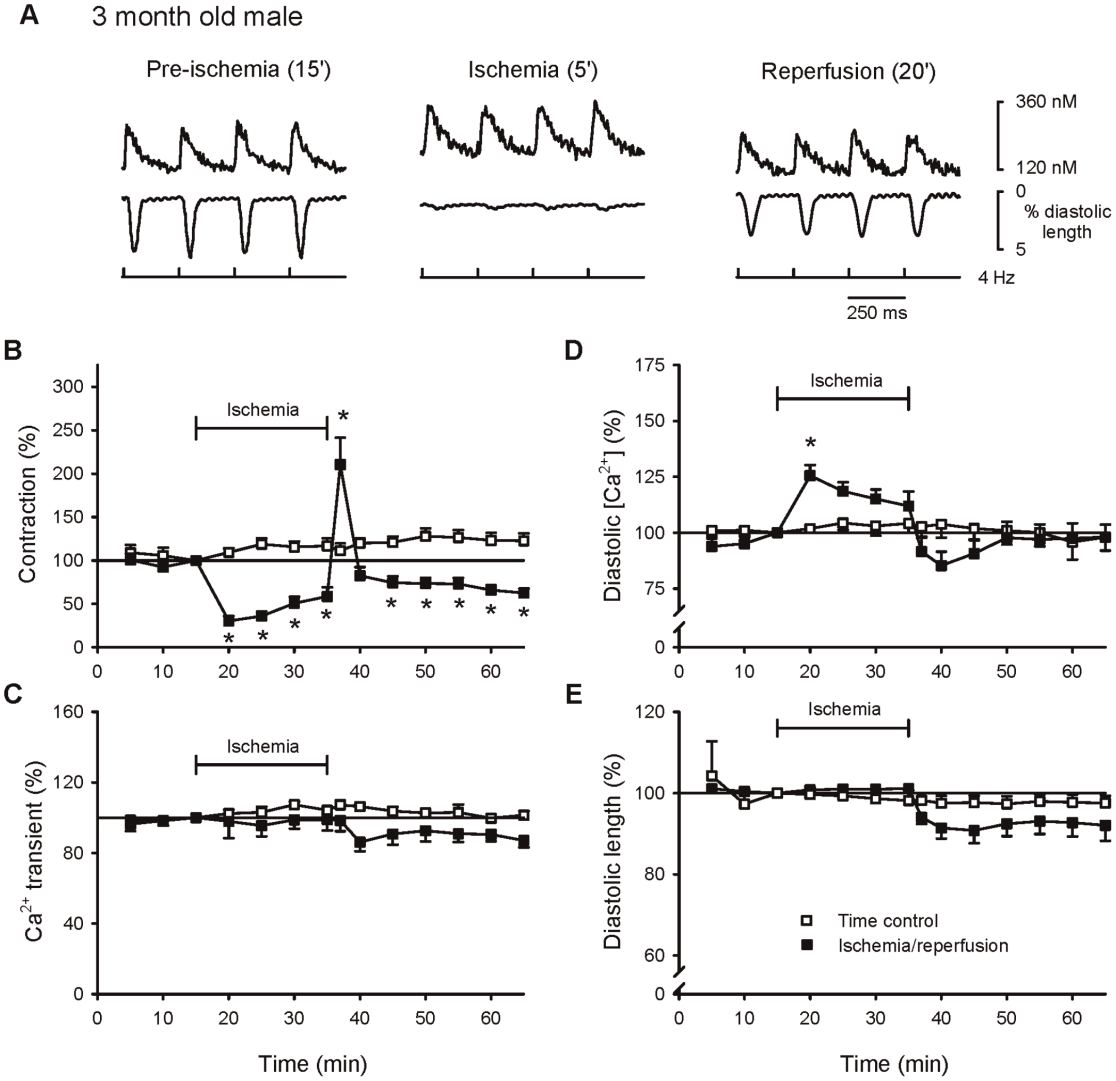
Ischemia inhibited contractions, enhanced diastolic Ca^2+^ loading, and promoted post-ischemic contractile dysfunction in myocytes from young adult male rats. Cells were paced at a frequency of 4 Hz for 15 minutes in normal Tyrode’s buffer, exposed to simulated ischemia for 20 minutes and reperfused with normal Tyrode’s for 30 minutes (filled squares). Time control cells were paced for the same length of time in normal Tyrode’s buffer only (open squares). **A.** Representative examples of Ca^2+^ transients (top) and contractions (bottom) recorded prior to ischemia, after 5 minutes of exposure to ischemia and after 20 minutes of reperfusion. **B.** Mean (± SEM) peak contractions recorded at 5 minute intervals throughout the experimental protocol. **C.** Mean amplitudes of Ca^2+^ transients recorded during these experiments. Mean levels of diastolic Ca^2+^ (**D**) and diastolic cell length (**E**) recorded throughout the protocol. In all cases, responses were normalized to values recorded after 15 minutes of stimulation, prior to exposure to ischemia. The * denotes significantly different from time control (p<0.05; n = 5 time control cells and 12 cells exposed to ischemia and reperfusion).

To determine whether responses of individual cardiomyocytes to myocardial ischemia were influenced by the sex of the animal, contractions and underlying Ca^2+^ transients were compared throughout ischemia and reperfusion in myocytes from young adult male and female rats. Figure 1A shows Ca^2+^ transients (top) and contractions (bottom) recorded from a male myocyte at selected time points throughout an experiment. Figure 1B–C shows mean peak contractions and Ca^2+^ transients recorded from male myocytes during ischemia and reperfusion compared to responses in time controls. Data were normalized to values recorded after 15 minutes of pacing at 4 Hz to facilitate comparisons between groups. Contractions were essentially abolished by ischemia but recovered with an overshoot immediately upon reperfusion (Figure 1B). However, peak contractions remained smaller than time controls with continued reperfusion (Figure 1B). In contrast, ischemia and reperfusion had no effect on Ca^2+^ transients throughout the experiment (Figure 1C). Ischemia caused a marked increase in diastolic Ca^2+^ and this recovered upon reperfusion (Figure 1D). Reperfusion also was associated with a modest degree of hypercontracture (Figure 1E). There were no signs of spontaneous activity in ischemia or reperfusion in this group (not shown). However, Trypan blue staining revealed that 38% of male myocytes were trypan blue positive by the end of the reperfusion period. Thus, male myocytes exposed to ischemia exhibited increased diastolic Ca^2+^, along with post-ischemic contractile dysfunction (stunning) and reduced viability in reperfusion.

**Figure 2:**
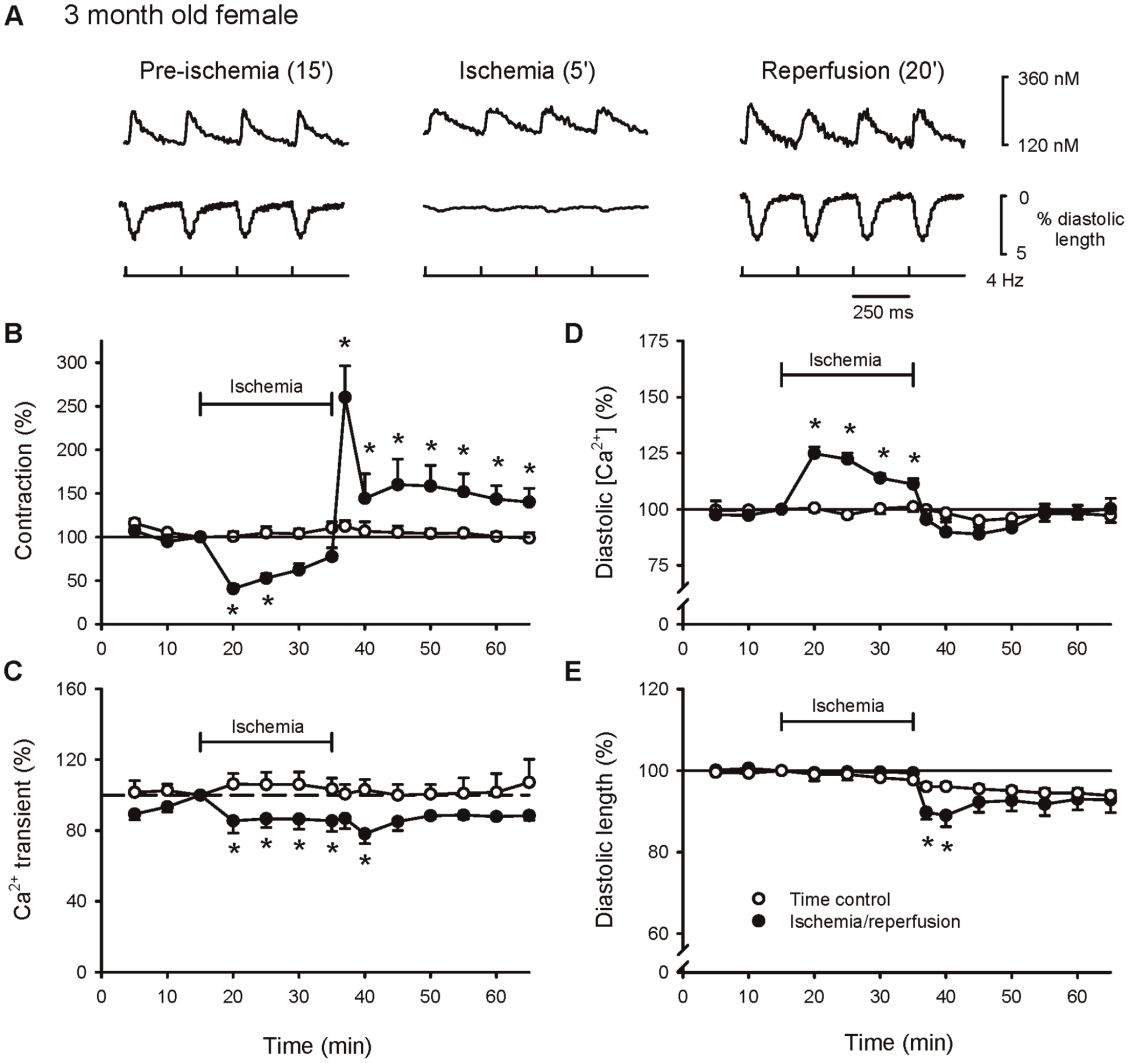
In contrast to males, ischemia inhibited Ca^2+^ transients and promoted recovery of contractile function in reperfusion in myocytes from young adult female rats. Myocytes were stimulated at 4 Hz for 15 minutes, exposed to ischemia for 20 minutes and reperfused for 30 minutes (filled circles). Cells that served as time controls were paced for 65 minutes without exposure to ischemia (open circles). **A.** Examples of Ca^2+^ transients (top) and contractions (bottom) recorded from female myocytes at specific times during an experiment. **B.** Mean amplitudes of contractions in females cells exposed to ischemia and reperfusion compared to time controls. **C.** Average amplitudes of Ca^2+^ transients recorded throughout the experiment. Mean levels of diastolic Ca^2+^ (**D**) and diastolic cell length (**E**) recorded throughout the experimental protocol. Responses were normalized to values recorded after 15 minutes of stimulation. The * denotes significantly different from time control (p<0.05; n = 9 time control cells and 10 cells exposed to ischemia and reperfusion).

Parallel ischemia and reperfusion experiments were then performed in myocytes from young adult females. Figure 2A shows representative Ca^2+^ transients (top) and contractions (bottom) in ventricular myocytes from female rats. Mean data show that contractions decreased significantly in ischemia, but recovered upon reperfusion and exceeded time controls throughout reperfusion (Figure 2B). Peak Ca^2+^ transients declined in ischemia and early reperfusion in the female group compared to time control cells (Figure 2C). Diastolic Ca^2+^ levels rose in ischemia and recovered in reperfusion (Figure 2D). Female cells initially exhibited hypercontracture in reperfusion, but this quickly recovered with continued reperfusion (Figure 2E). Spontaneous activity did not occur in either ischemia or reperfusion (not shown). Interestingly, all myocytes from young female rats survived exposure to ischemia and reperfusion.

**Figure 3:**
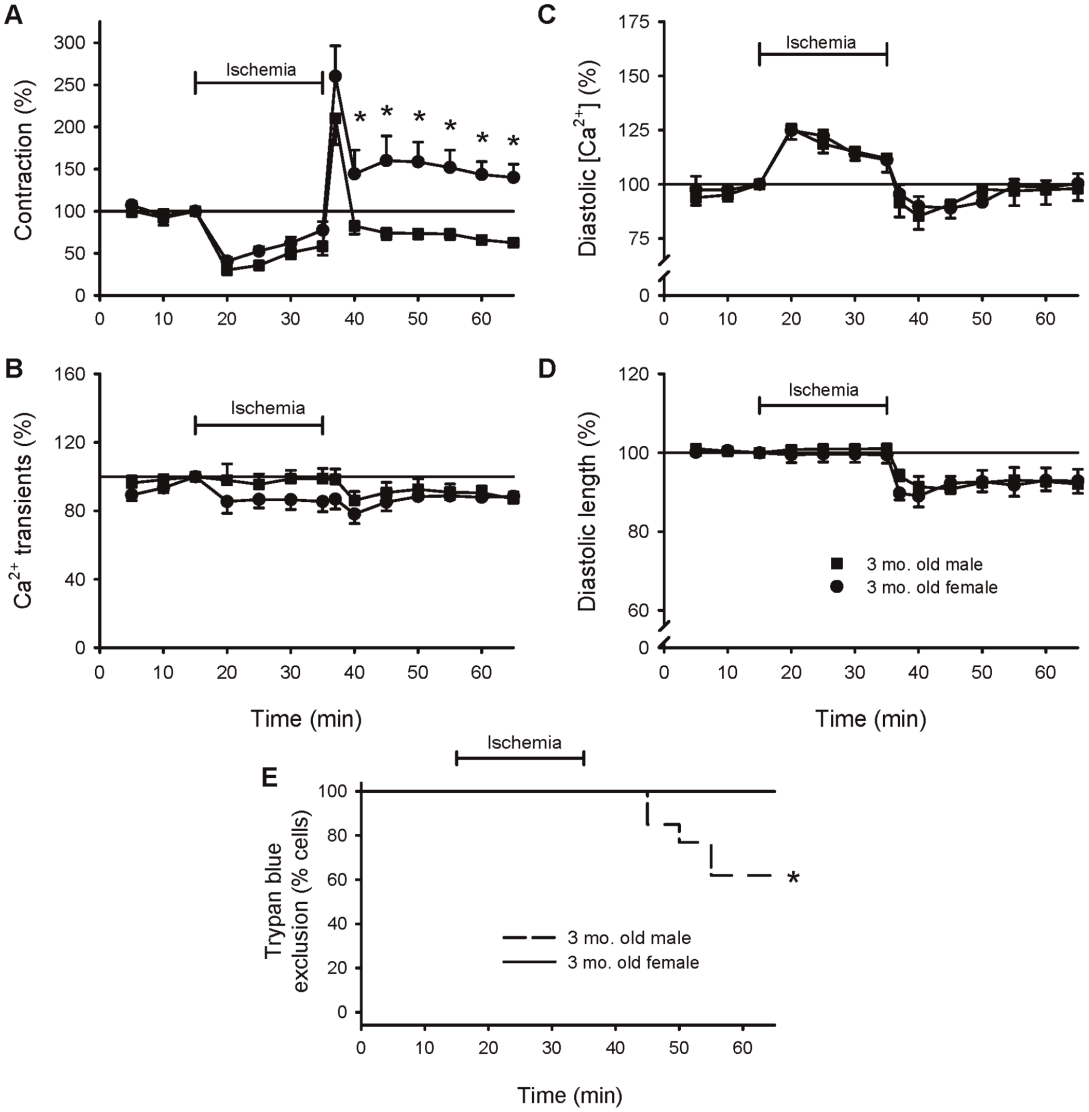
Myocytes from female rats exhibited less ischemia and reperfusion injury than cells from males. The protocol is as described in the legends to Figures 1 and 2. **A.** Mean magnitudes of contractions in male (filled squares) and female (filled circles) myocytes exposed to ischemia and reperfusion. **B.** Mean peak Ca^2+^ transients recorded from male and female myocytes at 5 minute intervals throughout exposure to ischemia and reperfusion. Average levels of diastolic Ca^2+^ (**C**) and resting myocyte length (**D**) recorded throughout the experimental protocol in cells from males and females. In all cases, data were normalized to values recorded after 15 minutes of stimulation. **E.** Survival curves illustrating the viability of male (dashed line) and female (solid line) cells at 5 minute intervals throughout the experimental protocol. The * denotes significantly different from young adult male value (p<0.05; n = 12 male cells and 10 female cells).

To determine whether responses to ischemia and reperfusion differed significantly between the sexes, contractions and underlying Ca^2+^ transients were directly compared as shown in Figure 3. Peak contractions were reduced by ischemia to a similar degree in cells from males and females (Figure 3A). However, while peak contractions were reduced throughout most of reperfusion in male myocytes, contractions in female cardiomyocytes actually fully recovered and were significantly larger than males in reperfusion (Figure 3A). Peak Ca^2+^ transients (Figure 3B) and diastolic Ca^2+^ levels (Figure 3C) were similar in male and female myocytes during ischemia and reperfusion, and the degree of hypercontracture in reperfusion did not differ between the two groups (Figure 3D). In contrast, exposure to Trypan blue revealed a significant difference in cell viability between the sexes. Although all myocytes in the young female group excluded trypan blue after exposure to ischemia and reperfusion, cell viability declined in reperfusion in the young male group and this sex difference was statistically significant (p<0.05). Taken together, these findings showed that myocytes from young adult females were resistant to ischemia and reperfusion injury, while myocytes from young adult males were not.

### Aging Disrupts Cardiomyocyte Ca^2+^ Handling and Exacerbates Ischemia and Reperfusion Injury in Female Myocytes

The next series of experiments determined whether ischemia and reperfusion injury in myocytes from female rats was exacerbated by the aging process. Baseline physical characteristics of the aged rats are compared to the younger animals in Table 1. The aged female rats were significantly older and heavier than the young adult females (Table 1). Interestingly, ventricular myocytes from aged females were 30% longer than myocytes from younger females (Table 1). However, peak contractions and Ca^2+^ transients were similar in cells from young and aged females prior to exposure to ischemia and reperfusion (Table 1).

**Figure 4:**
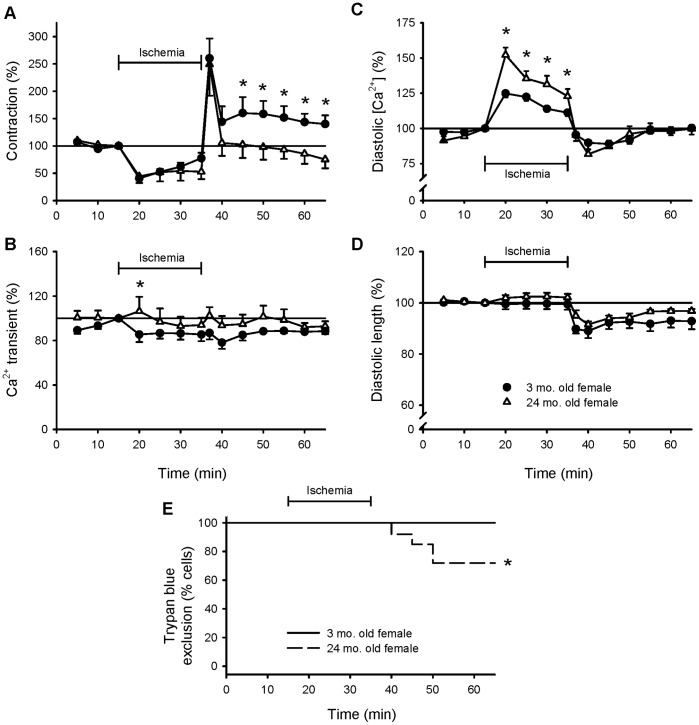
Aging promoted Ca^2+^ accumulation in ischemia and abolished beneficial effects of female sex on contractile function and cell viability. The experimental protocol is described in the legends to Figures 1 and 2. **A.** Mean amplitudes of contractions in young adult (filled circles) and aged female (open triangles) cells throughout exposure to simulated ischemia and reperfusion. **B.** Average peak Ca^2+^ transients recorded from young adult and aged female myocytes at 5 minute intervals throughout the experimental protocol. Mean levels of diastolic Ca^2+^ (**C**) and resting myocyte length (**D**) recorded at 5 minute intervals during the experiments in cells from young adult and aged females. All data were normalized to values recorded after 15 minutes of stimulation in the absence of ischemia. **E.** Survival curves illustrating the viability of young adult (solid line) and aged female (dashed line) cells reported at 5 minute intervals throughout the experiment. The * denotes significantly different from young adult female value (p<0.05; n = 10 young adult female cells and 8 aged female cells).

To determine whether ischemia and reperfusion injury in myocytes from female rats was exacerbated by aging, responses were compared in myocytes from young adult and aged female rats. Ischemia reduced peak contractions in both groups (Figure 4A). However, while contractions fully recovered upon reperfusion in the young group, contractions did not fully recover in the aged group (Figure 4A). Ca^2+^ transients were similar during most of ischemia and reperfusion in both groups (Figure 4B). Interestingly, aging augmented the rise in diastolic Ca^2+^ that occurred in ischemia (Figure 4C). The degree of hypercontracture in reperfusion was similar in the two groups (Figure 4D). However, even though all young female myocytes remained viable throughout ischemia and reperfusion, almost 30% of the aged myocytes were trypan blue positive by the end of reperfusion (p<0.05; Figure 4E). These results show that aging promoted Ca^2+^ loading in ischemia and abolished the beneficial effect of female sex on cell viability and contractile function in reperfusion.

**Figure 5:**
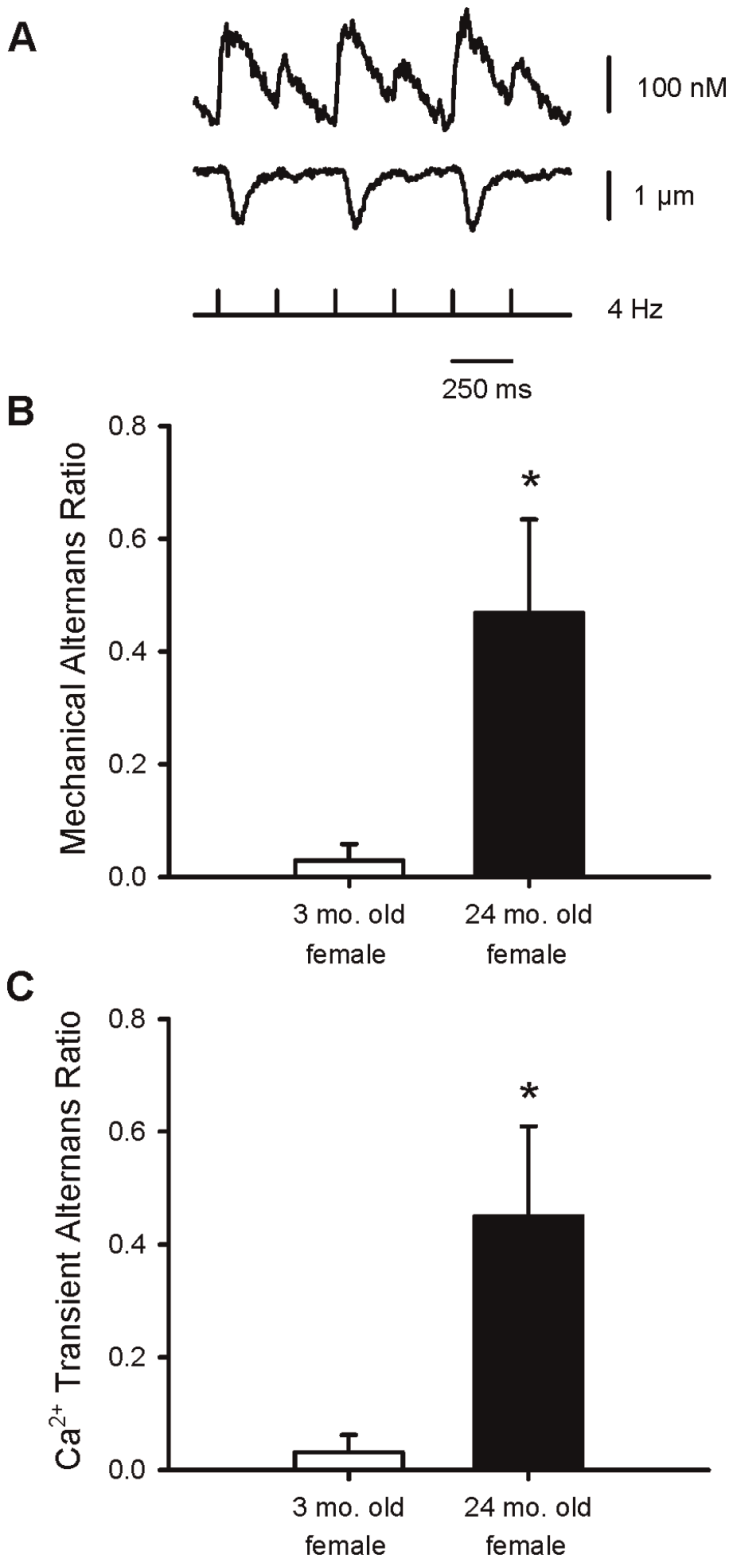
Aging was associated with the appearance of mechanical and Ca^2+^ transient alternans in ischemia in myocytes from female rats. **A.** Representative example of Ca^2+^ transient alternans (top) and mechanical alternans (bottom) ratios recorded in an aged female myocyte during ischemia. The occurrence of alternans was quantified as an alternans ratio with the following formula: alternans ratio  = 1 - S/L, where S  =  the amplitude of the small beat and L =  the amplitude of the large beat. Both the mechanical alternans ratio (**B**) and the Ca^2+^ transient alternans ratio (**C**) were significantly higher in myocytes from aged females than in young adult females. The * denotes significantly different from young adult female value (p<0.05; n = 10 young adult female cells and 7 aged female cells).

As aging was associated with increased Ca^2+^ loading in ischemia, this could promote abnormal activity and spontaneous Ca^2+^ release in ischemia and reperfusion. There was no evidence of spontaneous activity in ischemia or reperfusion in myocytes from aged rats, as in the younger adult animals (data not shown). However, ischemia did induce mechanical and Ca^2+^ transient alternans in myocytes from aged female rats, but not in cell from the younger female animals (Figure 5A). These responses were quantified with an alternans ratio as described in the methods section. Results showed mechanical (Figure 5B) and Ca^2+^ transient alternans ratios (Figure 5C) were increased dramatically in aged myocytes when compared to the younger group. Together, these results show that aging disrupted cardiomyocyte Ca^2+^ handling in myocytes from female hearts.

### OVX Exacerbated Detrimental Effects of Ischemia and Reperfusion in Isolated Ventricular Myocytes

To determine whether OVX would augment ischemia and reperfusion injury at the cellular level, responses of cardiomyocytes from sham-operated and OVX female rats were compared throughout ischemia and reperfusion. Physical characteristics of the sham-operated and OVX rats used in this study are shown in Table 2. The sham and OVX rats were similar in age, but OVX rats were almost 30% heavier (Table 2). OVX caused significant uterine atrophy in all animals, as shown by a striking decrease in uterine wet weight (Table 2). Uterine dry weights were also significantly lower in OVX animals (values decreased from 87.9±7.5 mg in sham to 8.2±1.3 mg in OVX; p<0.05). Interestingly, peak contractions and Ca^2+^ transients were larger in cells from OVX rats when compared sham-operated controls prior to exposure to ischemia and reperfusion (Table 2). OVX also increased diastolic Ca^2+^ under basal conditions (values increased from 105.0±5.0 in sham to 124.6±6.0 nM in OVX; p<0.05).

**Table 2:** Baseline Characteristics: Experiments with Sham-operated and OVX Female Rats.

Parameter	Sham-operated (n)	OVX (n)
Age (days)	165.2±7.4 (11)	179.8±9.3 (6)
Body weight (g)	182.0±5.7 (11)	234.3±7.0* (6)
Uterine wet weight (g)	0.460±0.04 (11)	0.039±0.01* (6)
Myocyte length (µm)	88.91±2.4 (7)	83.4±1.6 (7)
Contraction (%)	2.4±0.2 (7)	4.3±0.7* (7)
Ca^2+^ transient (nM)	92.5±4.7 (7)	111.0±5.1* (7)

Values shown are the means ± SEM. The value of "n" shown beside each number in brackets represents the number of animals or myocytes. Values represent peak contractions and Ca^2+^ transients (expressed as % resting cell length) recorded prior to ischemia in myocytes paced at 4 Hz.

* Denotes significantly different from sham-operated control animal, p<0.05.

**Figure 6:**
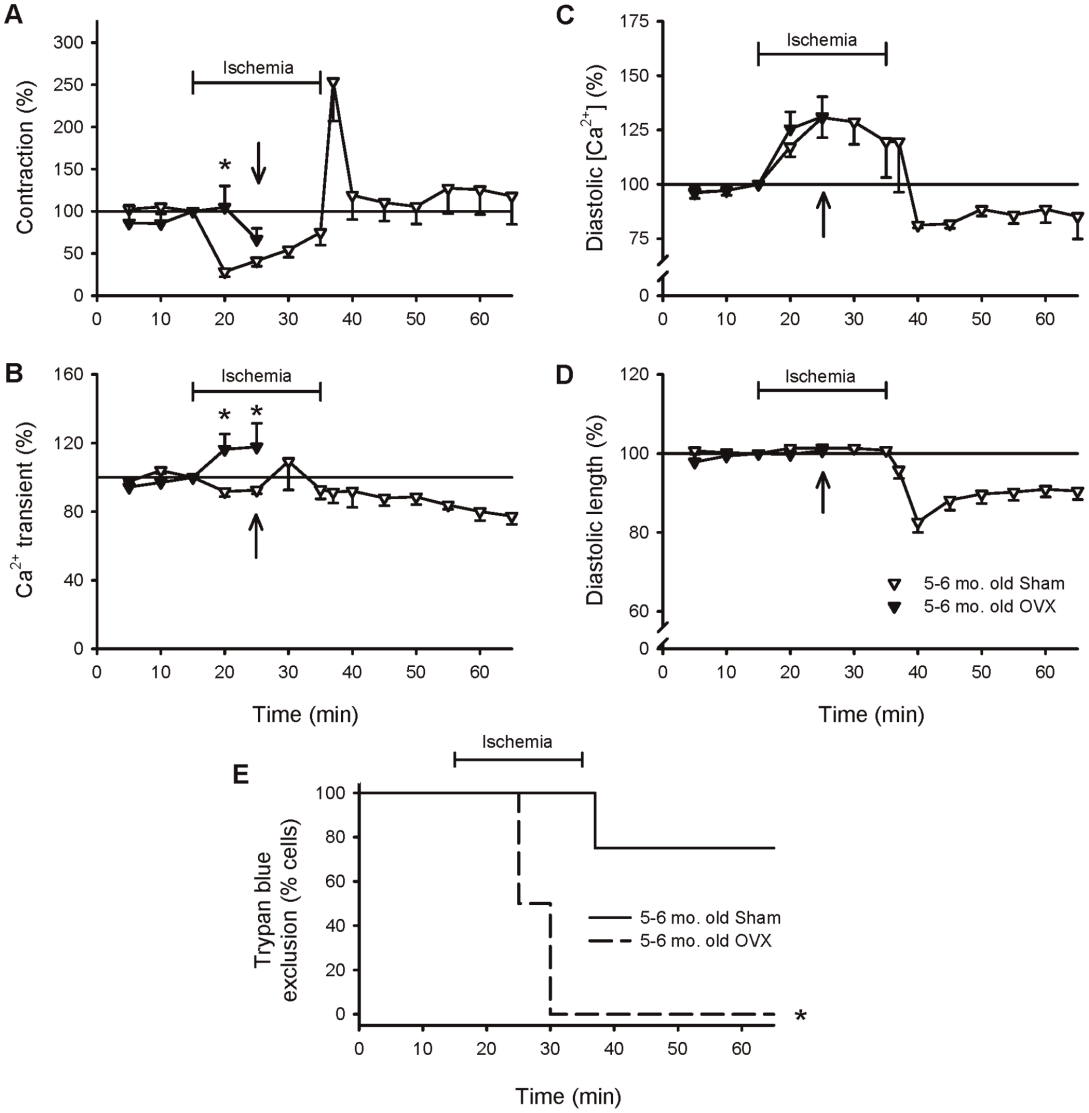
OVX increased contractions and Ca^2+^ transients in ischemia and none of the OVX myocytes were viable after 15 minutes of ischemia. The experimental protocol is described in the legends to Figures 1 and 2. **A**. Mean peak contractions recorded in myocytes from sham-operated (open inverted triangles) and OVX (filled inverted triangles) female rats throughout the protocol. The arrow indicates the last time point where OVX data could be collected because all OVX myocytes were trypan blue positive after 15 minutes of ischemia. **B**. Mean peak Ca^2+^ transients in sham and OVX myocytes recorded throughout the experiment. The average levels of diastolic Ca^2+^ (**C**) and diastolic cell length (**D**) in cells from sham and OVX females. Data were normalized to values recorded after 15 minutes of stimulation. **E**. Survival curves illustrating the viability of sham (solid line) and OVX female (dashed line) cells throughout the experiment. The * denotes significantly different from sham-operated control values (p<0.05; n = 7 sham cells and 7 OVX cells).

**Figure 7:**
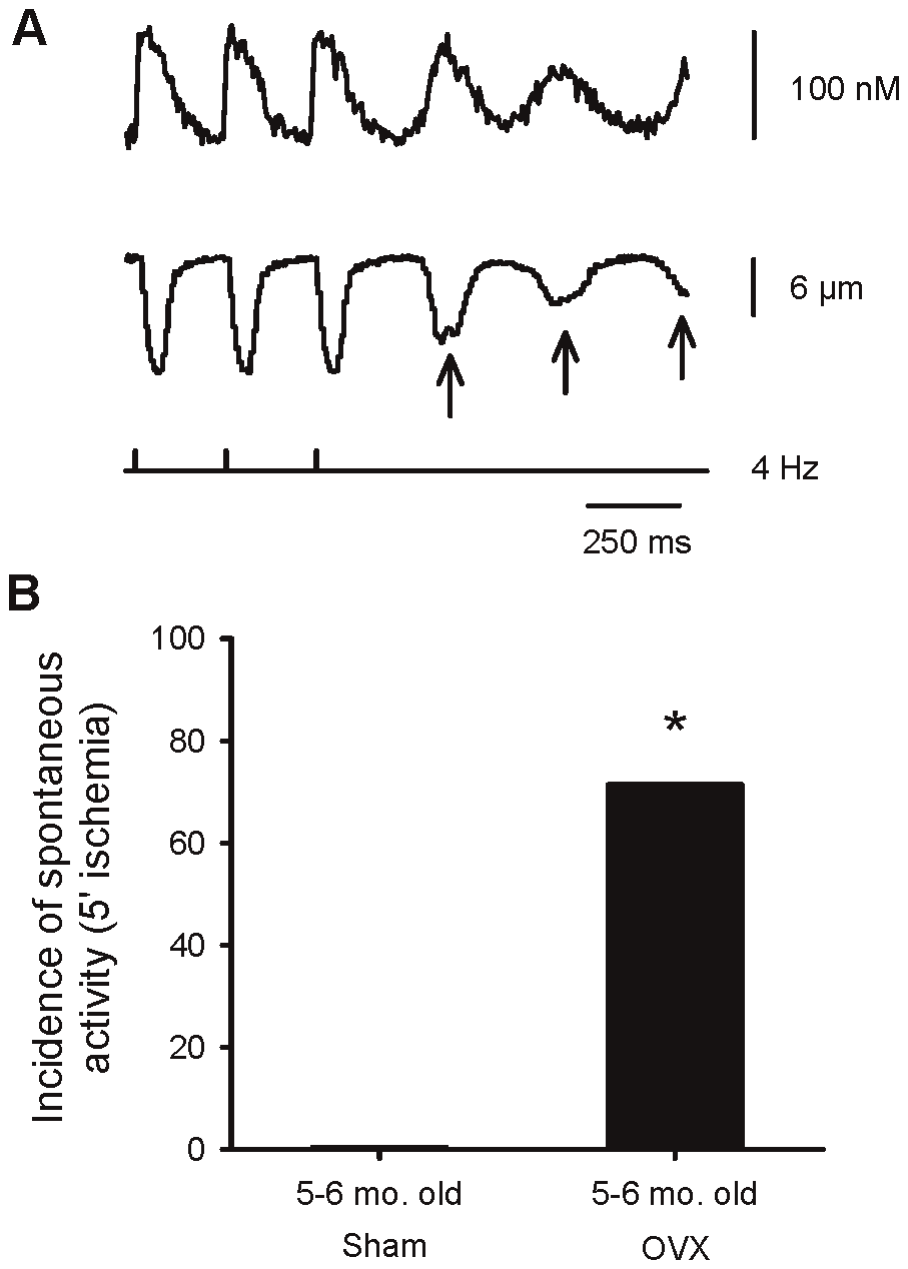
OVX promoted spontaneous contractions and Ca^2+^ transients in ischemia when compared to sham-operated controls. Cells were paced with trains of 20 pulses, delivered at a frequency of 4 Hz, followed by a 2.5 seconds delay to observe spontaneous activity. **A.** Representative examples of stimulated and spontaneous Ca^2+^ transients (top) and contractions (bottom) recorded in an OVX female myocyte after 5 minutes of exposure to ischemia. The first three beats are stimulated beats and the spontaneous responses are shown by the arrows. **B**. Spontaneous activity (Ca^2+^ transients and contractions) occurred in 71.4% of OVX cells. In contrast, spontaneous activity was not observed in myocytes from sham-operated animals. The * denotes significantly different from values in sham-operated females (p<0.05; n = 7 sham cells and 7 OVX cells).

To determine whether OVX modified ischemia and reperfusion injury, responses were compared in myocytes from sham and OVX rats. Note that none of the OVX myocytes were viable after 15 minutes of ischemia (Figure 6A–D, arrows), so effects late in the protocol were not available. While contractions declined in ischemia in sham controls, contractions remained large in the initial ischemic period in OVX cells (Figure 6A). Ca^2+^ transients also increased in ischemia in OVX cells, but not in sham controls (Figure 6B). However, the increase in diastolic Ca^2+^ levels in ischemia was similar in sham and OVX myocytes (Figure 6C) and diastolic length did not differ between the two groups (Figure 6D). Figure 6E shows that OVX dramatically reduced the ability of cardiomyocytes to tolerate ischemia. While most sham myocytes remained viable following exposure to ischemia and reperfusion, none of the OVX myocytes remained viable beyond 15 minutes of ischemia and this difference was statistically significant (p<0.05). Furthermore, 71% of OVX myocytes showed spontaneous Ca^2+^ release and contractions in ischemia, as shown in the example in Figure 7A. By contrast, spontaneous activity did not occur in myocytes from sham-operated controls (Figure 7B). These results show that OVX disrupted Ca^2+^ homeostasis and abolished the beneficial effects of female sex on cell viability in individual cardiomyocytes.

## Discussion

This study determined whether ventricular myocytes from young adult females were more resistant to ischemia and reperfusion injury than cells from males and whether ischemia and reperfusion injury in female myocytes was exacerbated by aging or by long term OVX. Results showed that responses of isolated myocytes to ischemia and reperfusion differed between the sexes. While ischemia reduced peak contractions and increased intracellular Ca^2+^ equally in myocytes from males and females, cells from males exhibited a profound reduction in post-ischemic contractile function but cells from females did not. In addition, all female myocytes remained viable during ischemia and reperfusion, but 38% of male myocytes were trypan blue positive in reperfusion. Interestingly, age abolished the beneficial effects of female sex on cell viability and contractile function. Age also augmented the rise in intracellular Ca^2+^ levels in ischemia and this was associated with a marked increase in the occurrence of alternans. OVX also modified myocyte responses to ischemia and reperfusion. Contractions and Ca^2+^ transients were larger in cells from OVX females than in cells from sham controls in the initial ischemic period and this was accompanied by an increase in the incidence of spontaneous activity. Furthermore, none of the myocytes from OVX animals were viable by 15 minutes of ischemia. Thus, ventricular myocytes from young adult males and aged or OVX females are more susceptible to injury following ischemia and reperfusion than cells from young adult females.

To our knowledge, this is the first study to demonstrate sex differences in the responses of individual ventricular myocytes to ischemia and reperfusion injury. While myocytes from male rats exhibited a marked reduction in contractile function throughout reperfusion, cells from females recovered fully and actually exceeded values recorded in time control cells. Previous studies in Langendorff-perfused hearts have shown that contractile function recovers more fully in reperfusion in females than in males [14], [16]–[20], although this has not been seen in all studies [22]. However, in intact hearts effects of estrogen on both the myocardium and the vasculature could contribute to cardioprotection in the setting of myocardial ischemia and reperfusion [11]. Our study is important as it shows that the improvement in recovery of myocardial contractile function in reperfusion in females is attributable, at least in part, to an increase in the ability of individual ventricular myocytes to contract.

As cardiac contractions are proportional to the magnitude of the Ca^2+^ transient [37], one explanation for the increased contractions in female myocytes during reperfusion is an increase in the size of the Ca^2+^ transient. However, our results show that this is not the case. Peak Ca^2+^ transients were not affected by ischemia and reperfusion. This result provides evidence that myofilament Ca^2+^ sensitivity increases in reperfusion in myocytes from young adult females. This contrasts with our observations in males, where reperfusion was associated with normal Ca^2+^ transients and a sustained reduction in peak contractions as shown previously [32], [33]. This post-ischemic decrease in contractile function (also called stunning) is thought to be due, at least in part, to degradation of troponin I, which leads to a reduction in myofilament Ca^2+^ sensitivity [38]. Our study shows that myocytes from young females do not exhibit stunning following myocardial ischemia and suggest that the underlying mechanism is an increase in myofilament Ca^2+^ sensitivity in reperfusion.

We also found that myocytes from young female were resistant to ischemia and reperfusion injury. Although 38% of the male myocytes were trypan blue positive in reperfusion, all the myocytes from 3 month-old female hearts resisted trypan blue stained throughout ischemia and reperfusion. This intrinsic resistance of myocytes to ischemia and reperfusion injury can explain the smaller infarcts and lower LDH release reported in female hearts following ischemia and reperfusion [17], [20], [23]–[25]. One mechanism that has been implicated in the loss of viability following ischemia and reperfusion is intracellular Ca^2+^ overload [39]. However, we found that intracellular Ca^2+^ levels rose during ischemia and declined in reperfusion to the same extent in myocytes from males and females. Thus, an important finding in our study is that the resistance of female myocytes to ischemia and reperfusion injury is not linked to sex differences in intracellular Ca^2+^ loading, because intracellular Ca^2+^ levels are similar in the two groups. Sex differences in other pathways may explain the resistance of female myocytes to ischemia and reperfusion injury. For example, sex differences in activation of apoptotic pathways and/or in the generation and regulation of reactive oxygen species may play a role in cardioprotection in the setting of ischemia and reperfusion [23], [40], [41].

To our knowledge, this is the first study to examine the impact of ischemia and reperfusion on Ca^2+^ homeostasis and contractile function in ventricular myocytes from aged female animals. A key finding in the present study was that age abolished beneficial effects of female sex on cell viability and contractile function in reperfusion. We found that, unlike young adult female cells, myocytes from 24 month-old females exhibited reduced cell viability and post-ischemic contractile dysfunction with no change in peak Ca^2+^ transients in reperfusion. Previous studies reported that recovery of contractile function is impaired in aged female hearts compared to younger hearts [20], [42], [43]. Our data demonstrate that post-ischemic contractile in the aging female heart is due contractile dysfunction at the level of the individual myocytes. Furthermore, the data presented here demonstrate that contractile dysfunction was not due to a decrease in size of the Ca^2+^ transient available to activate contraction. Thus, the aging process abolishes beneficial effects of female sex on these indices of myocardial damage.

Aging also resulted in diastolic Ca^2+^ accumulation and the development of alternans in ischemia in female myocytes, as reported in myocytes from aged male rats [33]. Elevated levels of intracellular Ca^2+^ are implicated in many detrimental effects of ischemia and reperfusion [39]. Our observation that aged female cells accumulate substantially more Ca^2+^ in ischemia than younger cells provides a mechanism that explains the increased sensitivity of aged female hearts to ischemia and reperfusion injury [20], [42,42]. The age-related decrease in activity and expression of sarco(endo)plasmic reticulum (SR) Ca^2+^-ATPase (SERCA) that has been well documented in aging male hearts [44] may also occur in aging female hearts. This would impair SR Ca^2+^ sequestration and disrupt intracellular Ca^2+^ regulation as shown in this study and previously [45]. Inhibition of SERCA in young adult hearts gives rise to cellular alternans [46], so reduced SERCA activity also may underlie the occurrence of alternans in the aging female heart.

Previous studies in rats have shown that serum estradiol levels decline by more than 60% between the ages of 3 mos and 14 mos [47], [48]. This suggests that cardioprotective effects of female sex may decline with age in response to a reduction in circulating estradiol levels. Indeed, we found that removal of ovarian estrogen through OVX exacerbated adverse effects of ischemia. In contrast to sham cells, contractions showed little decline in cells from OVX animals during the initial ischemic period. Furthermore, peak Ca^2+^ transients, which were larger than sham even prior to ischemia as shown in the present study and previously [49]–[53], actually increased significantly upon exposure to ischemia. Ischemia also increased the incidence of spontaneous Ca^2+^ transients and contractions, and this was followed by the uptake of trypan blue dye within 15 minutes of exposure to ischemia. The larger Ca^2+^ transients and spontaneous Ca^2+^ release in observed in OVX cells in ischemia suggests that SR Ca^2+^ load is much higher in OVX myocytes than in sham controls. Indeed, we and others have shown that SR Ca^2+^ load is elevated by OVX even in the absence of ischemia [50], [53]. The present study indicates that OVX promotes Ca^2+^ overload in ischemia and leads to spontaneous release of SR Ca^2+^ and ultimately a loss of cell viability.

Our results indicate that intracellular Ca^2+^ dysregulation is a major mechanism that contributes to the increased sensitivity of OVX hearts to ischemia and reperfusion injury [15], [23], [25], [54]. Interestingly, Ca^2+^ dysregulation in OVX myocytes may help explain the increased propensity for reperfusion arrhythmias observed in aromatase knockout mice, which exhibit both estrogen suppression and testosterone elevation [14]. However, chronic aromatase deficiency also has been shown to improve cardiac functional performance and limit acute cardiomyocyte injury in reperfusion [14]. Thus, suppression of androgen-to-estrogen conversion at the tissue level also may offer inotropic benefit in the setting of myocardial ischemia and reperfusion.

In the present study, ovaries were removed at 3–4 weeks of age but the rats were 5–6 months of age when we examined responses to ischemia and reperfusion. This resulted in an OVX model characterized by long term estrogen withdrawal and the lack of exposure of the heart to normal pubertal systemic estrogen modeling. It is important to note that this model differs from the more gradual reduction in ovarian steroids that would be observed during menopause. This model also may result in hearts that are particularly susceptible to ischemia and reperfusion damage. Additional experiments with other time frames for estrogen deprivation could be explored in the future to address these issues. It is also important to note that the present study examined only acute responses of cardiomyocytes to ischemia and reperfusion. The impact of ovarian estrogen suppression on other factors that may contribute to chronic post-ischemic heart failure also would be interesting to evaluate.

We also found that both aging and OVX caused increased a large increase in body weight when compared to younger female rats. This increase in body weight would be expected to increase systemic volume load and this could promote cardiomyocyte hypertrophy. Indeed, increased volume load may contribute to the increase in cardiomyocyte length we observed in the aged group. However, cardiomyocyte length was not affected by OVX, which suggests that increased volume load does not lead to cellular hypertrophy in OVX hearts, at least over the time frame of our investigation. It is likely that an increase in adipose tissue contributes to the increase in body weight observed in aged and OVX animals, so adipose tissue is a potential non-gonadal source of estrogen in our study [55]. Nonetheless, estrogen is likely low in these models, as studies have shown that estrogen levels decline with age in the rat model [47], [48] and remain lower in OVX rats than in sham animals up to 6 months after OVX [56].

Peak contractions, Ca^2+^ transients, and cell length recorded prior to exposure to ischemia and reperfusion also were examined in this study. Basal contractions and Ca^2+^ transients were larger in myocytes from OVX rats compared to sham-operated controls, as we and others have previously reported [49]–[53]. Basal contractions and Ca^2+^ transients also did not decline with age in female rats and aged myocytes were hypertrophied, as reported in our previous study [57]. However, the present study showed that basal contractions and Ca^2+^ transients were similar in amplitude in myocytes from 3 month-old male and female rats, when cells were paced at 4 Hz in 1.8 mM Ca^2+^ buffer. Previous studies that have reported smaller contractions and Ca^2+^ transients in female rat cells when compared to males used slower pacing frequencies and/or lower external Ca^2+^ concentrations [58]–[60]. These observations suggest that sex differences in contractile function may be more prominent when Ca^2+^ loading is reduced by low pacing frequencies and/or reduced external Ca^2+^ concentrations.

In summary, the results of our study show that resistance to myocardial ischemia and reperfusion injury is present in individual ventricular myocytes from young female animals. Our study also showed that this was abolished by either advanced age or by long term removal of ovarian estrogen. Aging and OVX both caused dramatic changes in intracellular Ca^2+^ homeostasis in individual cardiac myocytes exposed to simulated ischemia. Intracellular Ca^2+^ dysregulation plays an important role in cardiovascular diseases, so our data indicate that beneficial actions of estrogen in the setting of myocardial ischemia are mediated, at least in part, by actions on Ca^2+^ handling mechanisms in the myocytes themselves. Sex hormone-regulated pathways within cardiac myocytes may be a fruitful area to explore for the identification of new targets for the treatment of ischemic heart disease in men and women.

## Supporting Information

Appendix S1 **Supplemental materials and methods.**
(DOCX)
