# Supplementary material for: Age and Ovariectomy Abolish Beneficial Effects of Female Sex on Rat Ventricular Myocytes Exposed to Simulated Ischemia and Reperfusion
Source: PLoS One. 2012 Jun 6;7(6):e38425. doi: 10.1371/journal.pone.0038425 (PMC3368849; doi:10.1371/journal.pone.0038425)
Supplement: Appendix S1 — Supplemental materials and methods. (DOCX) [file pone.0038425.s001.docx]

**APPENDIX S1**

**Supplemental Materials and Methods.**

**Ethics Statement.** Animal protocols were approved by the Dalhousie University Committee on Laboratory Animals (protocol No. 10-029) and were performed in accordance with the Canadian Council on Animal Care Guide to the Care and Use of Experimental Animals (CCAC, Ottawa, ON: Vol, 1, 2nd edition, 1993: Vol. 2, 1984). Animals were anesthetized with sodium pentobarbital prior to cell isolation and all efforts were made to minimize suffering.

**Animals.** Young adult (3-6 mos) Fischer 344 rats of both sexes were obtained from Charles River Laboratories (Saint-Constant, QC); aged (~24 mos) female Fischer 344 rats were obtained from the National Institute on Aging (NIA, Baltimore, MD). Some experiments used young adult female Fisher 344 rats that had undergone a bilateral OVX or sham operation at the Charles River Laboratory when the animals were 3-4 weeks of age. All animals were housed on a 12-hour light/dark cycle in cages fitted with micro-isolator lids located in the Animal Care Facility at Dalhousie University.

**Myocyte Isolation.** Rat ventricular myocytes were isolated as described previously [1]. Briefly, rats were anesthetized with an IP injection of sodium pentobarbital (220 mg/kg) plus heparin (3000 U/kg). The heart was perfused through the aorta for 5 min with oxygenated (100% O_2_) buffer (mM): 135.5 NaCl, 4 KCl, 1.2 KH_2_PO_4_, 1.2 MgSO_4_, 10 HEPES, 12 glucose, 200 µM CaCl_2_ (pH 7.4; 37^o^C). Next, the heart was perfused for an additional 5 min with Ca^2+^-free buffer followed by perfusion with Ca^2+^-free buffer plus 50 μM Ca^2+^, dispase II (0.1-0.15 mg/mL, Roche Diagnostics, Laval, QC) and collagenase (0.3-0.6 mg/mL, Worthington Type II, Lakewood, NJ) for 15-20 mins. Following digestion, the ventricles were removed, minced and stored at room temperature in high K^+^ buffer (mM): 80 KOH, 30 KCl, 3 MgSO_4_, 50 glutamic acid, 30 KH_2_PO_4_, 20 taurine, 0.5 EGTA, 10 HEPES, 10 glucose (pH 7.4 with KOH). Individual cells were dissociated by gentle agitation and the cell suspension was filtered with a 225 μm polyethylene filter. Only rod shaped myocytes with no obvious membrane damage and no signs of spontaneous activity were used in experiments. Uterine atrophy was confirmed in all experiments by post-mortem removal of uterine tissue from each sham and OVX animal. Wet and dry uterine weights were recorded and compared in sham and OVX rats.

**Contractions and Ca^2+^ transients.** Fura-2 AM (5 μM) was added to the myocyte suspension and allowed to load for 20 min in the dark at room temperature. Cells were then placed in a 1 ml optical grade glass-bottomed chamber on the stage of an inverted microscope (Nikon Eclipse, Model TE2000 S, Nikon Canada Inc., Mississauga, ON) and allowed to adhere for 15 mins. The dye was removed by superfusion of myocytes at 6 mL/min with oxygenated (95% O_2_, 5% CO_2_) normal Tyrode’s solution containing (mM): 126 NaCl, 20 NaHCO_3_, 0.9 NaH_2_PO_4_, 4 KCl, 0.5 MgSO_4_, 1.8 CaCl_2_, 5.5 glucose (pH 7.4, 37^o^C). Myocytes were stimulated with a pair of platinum electrodes and a Grass SD9 Stimulator (Grass Medical Instruments, Quincy, MA) set at two times the threshold voltage. The stimulation frequency was controlled by a Frederick Haer stimulator (Pulsar 6i, Frederick Haer & Co., Brunswick, ME). Cells were paced at 4 Hz with trains of twenty 3 ms pulses followed by a 2.5 s delay to allow recording of spontaneous activity if present.

In all experiments, contractions and Ca^2+^ transients were recorded simultaneously as described previously [1]. Intracellular Ca^2+^ levels were recorded with a DeltaRam fluorescence recording system from Photon Technology International (PTI, Birmingham, NJ). Data were acquired with Felix32 software (PTI). Fura-2 loaded myocytes were alternately excited at 340 and 380 nm and emitted light was measured at 510 nm at a sampling interval of 5 msec. Background fluorescence was recorded at the end of each experiment and was subtracted from the measured emissions. Unloaded cell shortening was measured at 120 Hz with a video edge detector (Model 105; Crescent Electronics, Sandy, UT). Cells were viewed with a CCD camera (model TM-640, Pulnix America) and displayed on a television monitor. The analog signals for cell shortening were converted to digital signals (Digidata 1322A, Molecular Devices, Foster City, CA) and stored on a computer for analysis. Simultaneous recordings of contractions and fluorescence were made by splitting the microscope light with a dichroic cube (Chroma Technology, Brattleboro, VT). Red light was sent to the closed circuit camera and video edge detection system, while the remaining light was delivered to the photomultiplier tube for fluorescence measurement.

**Simulated ischemia and reperfusion protocol.** Cells were equilibrated in normal Tyrode’s solution (6 mL/min) and control recordings were made for 15 min. Then cells were superfused for 20 min with a simulated ischemic Tyrode’s solution of the following composition (mM): 123 NaCl, 6 NaHCO_3_, 0.9 NaH_2_PO_4_, 8 KCl, 0.5 MgSO_4_, 20 Na-Lactate, and 1.8 CaCl_2_ (90% N_2_, 10% CO_2_; pH 6.8) [2]. During ischemia, a 90% N_2_, 10% CO_2_ gas phase was directed over the experimental chamber. Under these conditions, the *p*O_2_ of the ischemic Tyrode's solution declined rapidly by more than 90%, as reported by our group previously [3]. After exposure to ischemia, cells were reperfused with normal Tyrode’s solution for up to 30 min. At the end of each experiment, 0.5 to1 mL of Trypan blue dye (0.4%) was added to the experimental chamber as in previous studies [1,3]. Cells that absorbed trypan blue dye exhibited hypercontracture and sarcolemmal disruption. These trypan blue permeant cells no longer had intact cell membranes and were defined as non-viable myocytes [4].

Recordings were made at 5 min intervals throughout the protocol, with an additional recording at 2 min of reperfusion. Contractions were recorded for 20 sec at each interval but fluorescence recordings were made for only 8 sec to avoid photobleaching the Ca^2+^ indicator. For time control experiments, myocytes were exposed to normal Tyrode’s solution for 65 min without an ischemic period and recordings of contractions and Ca^2+^ transients were made at the time points described for the ischemia and reperfusion protocol. The ratio of fluorescence emission at 340 and 380 nm was converted to Ca^2+^ concentration with an *in vitro* calibration curve determined with known concentrations of Ca^2+^, as described in our previous studies [1,5]. The Ca^2+^ concentration measured with this approach is accurate over the range of pH values for the buffers used in this study, since the effect of pH on fura-2 within the physiological range is negligible [6]. Furthermore, we previously showed that calibration curves determined over the pH range used in this study were similar at Ca^2+^ concentrations between 100 nM and 1 μM [1,5].

**Data analysis.** Contractions and Ca^2+^ transients were analyzed with Clampfit 8.2 software (Molecular Devices). The last three contractions in the 20 pulse train were averaged; contraction amplitudes were measured as the difference between resting (diastolic) cell length and the peak of cell shortening. Peak Ca^2+^ transients were measured as the difference between systolic and diastolic Ca^2+^ concentrations. As with contractions, an average of the last three transients in the train of 20 stimulated beats was used to measure Ca^2+^ concentrations and Ca^2+^ transient amplitudes. In some experiments, abnormal Ca^2+^ release and contractions occurred. Spontaneous activity was defined as extra beats that occurred following the last stimulated beat in a train and the incidence of this activity was recorded in each experiment. Mechanical and Ca^2+^ transient alternans, defined as an alternating pattern of large and small beats during the stimulation train, also occurred in some experiments. Alternans were quantified with an alternans ratio calculated follows: alternans ratio = 1 - S/L, where S = the amplitude of the small beat and L= the amplitude of the large beat [1].

**Statistical Analysis**. The value of "n" represents the number of myocytes used in each experiment. No more than two myocytes from the same animal were used in any one experiment. Each myocyte was subjected to a single round of ischemia and reperfusion to avoid preconditioning the cells. Statistical analyses were performed with either Sigmaplot 8.1 or Sigmastat 3.1 (Systat Software Inc.). Data other than cell viability and incidence are presented as the mean ± SEM. Cell viability is shown as a Kaplan-Meier survival curve and differences in viability between groups were evaluated with a log rank test. The incidence of spontaneous activity was analyzed with a Fisher Exact test for χ2 contingency tables. Other differences between groups were evaluated with either a t-test or a two-way repeated-measures analysis of variance with Bonferroni correction. Student-Newman-Keuls or Tukey tests were used for post hoc comparisons. Differences between groups were considered statistically significant for values of p<0.05.

**Chemicals.** Fura-2 AM (Invitrogen, Burlington, ON) stock solution was prepared in anhydrous DMSO with a final concentration of 0.2% DMSO and aliquots were stored desiccated at -20^o^C until use. All other chemicals were purchased from Sigma-Aldrich (Oakville, ON).

**References:**

1. O'Brien JD, Howlett SE (2008) Simulated ischemia-induced preconditioning of isolated ventricular myocytes from young adult and aged Fischer-344 rat hearts. Am J Physiol Heart Circ Physiol 295: H768-H777.

2. Cordeiro JM, Howlett SE, Ferrier GR (1994) Simulated ischaemia and reperfusion in isolated guinea pig ventricular myocytes Cardiovasc Res 28: 1794–1802.

3. Ross JL, Howlett SE (2009) Beta-adrenoceptor stimulation exacerbates detrimental effects of ischemia and reperfusion in isolated guinea pig ventricular myocytes. Eur J Pharmacol 602: 364-372.

4. Stoddart MJ (2011) Cell viability assays: introduction. Methods Mol Biol. 2011;740:1-6.

5. Louch WE, Ferrier GR, Howlett SE (2002) Changes in excitation-contraction coupling in an isolated ventricular myocyte model of cardiac stunning. Am J Physiol Heart Circ Physiol 283: H800-H810.

6. Grynkiewicz G, Poenie M, Tsien RY (2006) A new generation of Ca^2+^ indicators with greatly improved fluorescence properties. J Biol Chem 260: 3440-3450, 1985.
